# An Exploration of the Viral Coverage of Mosquito Viromes Using Meta-Viromic Sequencing: A Systematic Review and Meta-Analysis

**DOI:** 10.3390/microorganisms12091899

**Published:** 2024-09-14

**Authors:** Shenglin Chen, Yuan Fang, Ryosuke Fujita, Emad I. M. Khater, Yuanyuan Li, Wenya Wang, Peijun Qian, Lulu Huang, Zhaoyu Guo, Yi Zhang, Shizhu Li

**Affiliations:** 1 National Key Laboratory of Intelligent Tracking and Forecasting for Infectious Diseases, National Institute of Parasitic Diseases, Chinese Center for Disease Control and Prevention, Chinese Center for Tropical Diseases Research, NHC Key Laboratory of Parasite and Vector Biology, WHO Collaborating Center for Tropical Diseases, National Center for International Research on Tropical Diseases, Shanghai 200025, China; chenshenglin0909@163.com (S.C.); fangyuan@nipd.chinacdc.cn (Y.F.); liyy@nipd.chinacdc.cn (Y.L.); wangwenya20220301@163.com (W.W.); yqjkzpj@163.com (P.Q.); llhuang@nipd.chinacdc.cn (L.H.); dayuguo1129@outlook.com (Z.G.); zhangyi@nipd.chinacdc.cn (Y.Z.); 2 School of Global Health, Chinese Center for Tropical Diseases Research, Shanghai Jiao Tong University School of Medicine, Shanghai 200025, China; 3 Laboratory of Sanitary Entomology, Faculty of Agriculture, Kyushu University, Fukuoka 819-0395, Japan; r-fujita@agr.kyushu-u.ac.jp; 4 Department of Entomology, Faculty of Science, Ain Shams University, Cairo 11566, Egypt; eikhater@yahoo.com

**Keywords:** metagenomic analysis, mosquito viromes, mosquito-borne viruses, meta-analysis

## Abstract

The aim of this review was to delve into the extent of mosquito virome coverage (proportion of viral reads) via meta-viromic sequencing and uncover potential factors of heterogeneity that could impact this coverage. Data sources were PubMed, Web of Science, Embase, Scopus, Science-Direct, Google Scholar, and the China National Knowledge Infrastructure. Pooled coverage was estimated using random-effects modeling, and subgroup analyses further reveal potential heterogeneous factors. Within the three mosquito genera studied, *Culex* exhibited the highest pooled viral coverage of mosquito viromes at 7.09% (95% CI: 3.44–11.91%), followed by *Anopheles* at 5.28% (95% CI: 0.45–14.93%), and *Aedes* at 2.11% (95% CI: 0.58–7.66%). Subgroup analyses showed that multiple processing methods significantly affected the viral coverage of mosquito viromes, especially pre-treatment of mosquito samples with saline buffer/medium and antibiotics prior to DNase/RNase treatment and removal of the host genome prior to RNA library construction. In conclusion, the results of this study demonstrate that the viral coverage of mosquito viromes varies between mosquito genera and that pre-treatment of mosquito samples with saline buffer/medium and antibiotics before DNase/RNase treatment and removing host genomes prior to RNA library construction are critical for the detection of RNA viruses in mosquito vectors using meta-viromic sequencing.

## 1. Introduction

Mosquitoes (Diptera: Culicidae) belong to an insect family comprising more than 3600 species [1]. Mosquito-borne viruses are estimated to cause more than 100 million cases of human disease annually [2]. Mosquitoes are common vectors of arboviruses, which can cause a number of major diseases, including dengue virus (DENV), Zika virus (ZIKV), yellow fever virus (YFV), West Nile virus (WNV), Japanese encephalitis virus (JEV), Chikungunya virus (CHIKV), Getah virus (GETV), and Banna virus (BAV) [3,4,5,6,7,8]. Mosquitoes may play a larger role in viral ecology and disease transmission than thought and should be regarded as an important surveillance target for the prevention and control of infectious diseases.

Viromes are structurally and functionally diverse, with varying host and environmental habitats. Generally, mosquito-borne virus detection is based on virus isolation [9] and polymerase chain reaction methods [10,11,12]. However, these conventional testing methods are time-consuming and labor-intensive in detecting low levels of mosquito viromes and are targeted assays [10,11,13]. With the continuous development of high-throughput sequencing technologies, metagenomic next-generation sequencing (mNGS) analyses provide a better way to understand the diversity and abundance of mosquito-borne pathogens and novel pathogens, especially since little was known about invertebrate and vertebrate viromes prior to the development of high-throughput sequencing technologies [14,15,16]. Metagenomic sequencing is particularly well suited for high-throughput viral sampling studies; this method does not require prior information of the organism to be identified and does not require specific primers or antibodies, making it the most unbiased means of detection available. The detection of new viruses is one of the main advantages of metagenomic sequencing over other detection methods [15,17].

Viral sequence analysis from metagenomic data in mosquito samples has broadened our understanding of viral abundance, diversity, evolution, and role in host biology [14,16]. Metagenomic analyses are valuable in avoiding missed detection of highly infectious and pathogenic viruses, as well as unknown viruses in different mosquito species, especially for the identification of hidden RNA viruses [15,16,17]. The most common mosquito-borne arboviruses whose genetic material is RNA are transmitted by two mosquito genera: *Aedes* and *Culex* [16,18,19]. RNA viruses are dominant in *Aedes* (e.g., YFV, ZIKV, DENV, CHIKV) [20] and *Culex* (e.g., Eastern equine encephalitis virus, Rift Valley fever virus, WNV) [19]. Within the genus *Aedes*, two specific species, *Aedes aegypti* [21] and *Aedes albopictus* [22], have been shown to be important vectors of globally important arboviruses, including DENV [23], YFV [18], ZIKV [24], and CHIKV [25]. *Culex* mosquitoes are vectors of many important pathogens, including WNV, JEV, and Sindbis virus (SINV) [7,26]. In addition, another important mosquito genus, *Anopheles*, is thought to be a vector for viruses such as JEV, BAV, and Kadipiro virus (KDV) [8,27,28], and the *Anopheles* virome appears to be dominated by RNA viruses (e.g., JEV, BAV) [29]. Therefore, understanding the application of meta-viromic sequencing technology in these three major mosquito genera has important practical significance, and the virome exploration in this study will focus on RNA library sequencing.

Virological surveillance of mosquitoes to recognize viruses with zoonotic disease potential is a significant tool to help improve preparedness and prevent outbreaks. Mosquito-borne virus surveillance based on high-throughput sequencing remains in its early stage, and the methodology for mosquito viromes using meta-viromic sequencing has not yet been harmonized. Understanding the heterogeneity factors in meta-viromic sequencing applications is crucial for optimizing coverage. Variations in mosquito pre-treatment, host genome removal, and bioinformatics pipelines warrant exploration, as do potential differences in virome coverage among mosquitoes of the same genus in varying climates. A systematic review and meta-analysis are essential to address these issues comprehensively and enhance the methodology of meta-viromic sequencing for improved mosquito-borne virus surveillance. However, to the best of our knowledge, no published systematic review and meta-analysis have explored the proportion (coverage) of mosquito viromes obtained via virome sequencing techniques. Thus, based on the above research objectives, this study aims to comprehensively analyze the coverage of mosquito viromes obtained based on meta-viromic sequencing and to provide a theoretical basis for further optimizing the methodology of meta-viromic sequencing technology and expanding its application so as to better implement mosquito-borne-virus surveillance.

## 2. Materials and Methods

### 2.1. Data Sources and Search Strategy

This systematic review and meta-analysis was conducted according to the Preferred Reporting Items for Systematic Reviews and Meta-analyses (PRISMA) 2020 Checklist [30]. A protocol was developed but was not uploaded to PROSPERO. The study focused on original research concerning mosquito viromes in mosquito pools, with a clear description of meta-viromic sequencing. Inclusion criteria encompassed (a) original research on mosquito viromes; (b) clear high-throughput sequencing methodology; (c) field-collected mosquitoes; (d) specific values for raw data reads provided in the included primary literature; and (e) specific values for viral sequence reads available in the original literature, either directly (in the text) or indirectly (in the Supplementary Materials). Specific values of raw data are the paired-end reads generated after post-library sequencing that have not been analyzed by the bioinformatics pipeline. In each of the included primary studies, the specific values of raw data are the total reads. Exclusion criteria included (a) meta-viromic studies of individual mosquitoes; (b) virological data from virus inoculation and infection in mosquitoes; (c) exclusion of RNA library construction sequencing; (d) the types of primary literature (reviews, commentaries, and conference papers); and (e) sequencing data obtained from databases rather than from the authors through high-throughput sequencing and first publication.

A literature search using Web of Science, Embase, Medline (via PubMed), Google Scholar, Science-Direct, Scopus, and the China National Knowledge Infrastructure (CNKI) did not include limitations on the date (from inception to 5 March 2024) or language to ensure that more studies were included. MeSH terms such as meta-viromic sequencing, mosquito virome, metatranscriptome sequencing, virome, mosquito, and relevant keyword combinations were employed. Chinese characters were used for retrieval in CNKI, and the main retrieval method was consistent with retrieval from the other sources. After duplicate removal using EndNote, two reviewers (SC, YF) independently screened the literature, and disputes were resolved through negotiation. The exhaustive search strategy is reported in Supplementary Materials (Supplementary File S1).

### 2.2. Data Extraction and Quality Assessment

Data extracted from individual studies included (a) sampling location, month or temperature of collection, and climate type; (b) mosquito sample preparation, RNA extraction, host genome removal, and bioinformatics pipeline details; and (c) sequencing platforms, mosquito genus levels, total reads, and viral reads. Raw data refer to the original data post-RNA library construction and sequencing. Two researchers (SC and YF) independently conducted data extraction, resolving disagreements through consensus with the third author (SL).

Based on the characteristics of this study, a customized grading system was developed to assess the quality of each study included in the review. Several published studies have used a similar scoring system to assess the quality of evidence for included articles [31,32,33,34]. We assigned each of the original studies included in the systematic review a grade for each of nine quality items: clarity of the research objective/question (was the research objective/question clearly described and stated?). If the information provided was insufficient to assign quality scores of the study (e.g., no description was given and no inferences could be drawn), it was deemed unable to judge. Details of the quality assessment of all included literature based on the customized scoring system of this study are as follows. In evaluating the “research question”, a score of 3 indicated full conformity between the original literature study’s purpose and the review study’s purpose, while a score of 2 signified partial conformity, and a score of 1 indicated complete inconsistency. With regard to “sampling location”, 3 points were awarded if there was a detailed description (latitude, longitude, and village) in the original literature, 2 points if only the city was mentioned without being precise about the villages, and 1 point if only the area where the study was carried out was indicated (at the national level). For “temperature description”, a score of 3 was assigned for a detailed temperature description, 2 for describing the sampling season, and 1 for mentioning only climatic zones. Three points were awarded for a specific description of the “sampling method” (sampling tool and sampling process), two points for mentioning only the sampling tool, and one point for not providing any description. A score of 3 was given if the original literature details the pre-treatment method of mosquito samples before DNase/RNase treatment and RNA extraction, a score of 2 for cursory descriptions where no details are provided, while a score of 1 is assigned if this method is not described. Three points were awarded if the RNA extraction methods provided a detailed description; two points were awarded if no details were provided; and one point was awarded if no description was provided. Additionally, a score of 3 was given if the steps to remove the host genome after RNA extraction and before library sequencing are described in detail, a score of 2 was given if they are briefly described or inferred, and a score of 1 was given if they are not described. Finally, for “sequencing platforms”, a score of 3 was given for a careful description of the platform and the sequencing instrument model, a score of 2 was given for providing only the platform, and a score of 1 was given for no description. Three authors (SC, YF, and SL) independently performed quality assessment of all included research and disagreements were resolved by discussion.

### 2.3. Data Analysis

Based on the characteristics of the data included in this study, the raw values of coverage (ratio of virus reads to total reads) were subjected to Freeman–Tukey double-arcsine transformation and thus met the normality test, and the random effects model was finally selected for the pooled analysis of coverage based on the results of the heterogeneity test. Meanwhile, given the significant variations in sample size, study design, species sampling location, climate type, and sampling time across studies, a random effects model was similarly required for the analysis [35]. This model was utilized to aggregate the coverage of mosquito viromes, with the assessment of heterogeneity among studies conducted using the Chi-squared test (Q test) and I-square statistic. Subgroup analysis was performed to elucidate the impact of potential explanatory factors, such as pre-treatment of mosquito samples (prior to RNA extraction), host genome removal (before RNA library construction), bioinformatics pipeline (database), sequencing read-length, climate types, and region (continents). Subgroup variables were categorized into saline buffer or medium, saline buffer or medium plus antibiotics, and no saline buffer/medium/antibiotics based on how the mosquito samples were pre-treated. Based on the characteristics of the original literature ultimately included, we divided the geographic regions based on continental level into Asia, the Americas (South and North America), and Europe.

The primary focus of this study lies in evaluating the coverage of the mosquito viromes, which represents the ratio of viral sequence reads obtained through blast comparison to the raw data post-library sequencing. As such, our analysis predominantly delves into the intricate bioinformatics processes of quality control, host genome removal, and sequence alignment. When amalgamating the findings from the 16 studies included, the key disparity in these bioinformatics procedures stemmed from the utilization of different databases (NCBI database and custom database). Additionally, we delved into exploring the impact of sequencing read-length (<150 bp and 150~300 bp) on the coverage of mosquito-borne viromes within *Aedes* mosquito pools across various climatic zones categorized as tropical, subtropical, and temperate.

Given the substantial raw data values in this study, the Freeman–Tukey double-arcsine transformation was predominantly employed for proportion calculations following a normality test and data transformation. Sensitivity analyses were conducted using a generalized linear mixed model (GLMM) with a logit transformation to mitigate potential inaccuracies associated with the double-arcsine transformation [36,37]. An evaluation of publication bias was undertaken for meta-analyses comprising four or more studies. Bias assessment involved the utilization of funnel plots and tests for funnel plot asymmetry based on methods proposed by Egger [38]. In instances where Egger’s test indicated bias, the Trim-and-Fill technique was employed to estimate the impact of missing studies on the meta-analysis outcomes [39]. Publication bias was further assessed through the implementation of the linear regression test of funnel plot asymmetry using the metabias (R package, R software version 4.1.0). All *p*-values were derived from two-sided tests, with a significance level set at *p* < 0.05. Heterogeneity levels were categorized as low (I^2^ ≤ 25%) or high (≥75%). The analyses were executed using the ‘meta’ package in R software version 4.1.0 (R Project for Statistical Computing, Vienna, Austria) (http://www.R-project.org, accessed on 8 September 2024).

## 3. Results

### 3.1. Search Results

**Figure 1:**
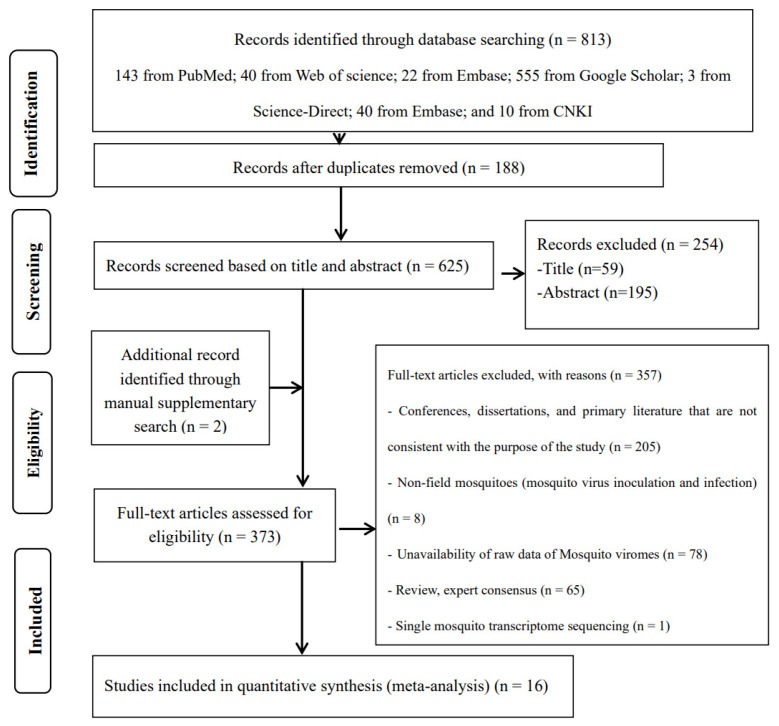
Preferred Reporting Items for Systematic Reviews and Meta-Analyses (PRISMA) flowchart of the study selection.

The search strategy identified a total of 625 studies, of which 16 met all selection criteria for meta-analysis (Figure 1). Of these 16 included original studies, 11 reported the viral coverage of mosquito viromes in mosquito pools of mixed genera, 7 reported the viral coverage of mosquito viromes in mosquito pools of *culex*, 8 reported the viral coverage of mosquito viromes in mosquito pools of *Aedes*, and only 4 reported the viral coverage of mosquito viromes in mosquito pools of *Anopheles*. Table 1 provides basic characteristics of the viral virome coverage in pools of mixed genera, *Culex*, *Aedes*, and *Anopheles*. These characteristics mainly include total reads (raw data), viral reads, and subgroup analysis variables. Table S1 presents details on climate type, pre-treatment of mosquito samples (before DNase/RNase treatment and RNA extraction), nucleic acid extraction (RNA), host genome removal (pre-library sequencing), sequencing platforms, bioinformatics analysis, mosquito genus levels, virus identification criteria, number of mosquitoes per pool (mean), and locations as documented in the original literature.

### 3.2. Quality Assessment

Table 2 outlines the quality scores for all 16 primary studies included. The literature incorporated in the study exhibited a low risk of bias concerning the research question, nucleic acid extraction (RNA), sampling method, and sequencing platforms. While five original studies omitted descriptions of temperatures or seasons during sampling [19,46,47,49,50], they were able to infer climatic zones based on sampling locations. Overall, these ambiguities in bias did not raise significant concerns regarding the relevance of mosquito virome coverage across the majority of studies.

### 3.3. The Viral Coverage of Mosquito Viromes in Mosquito Pools

**Figure 2:**
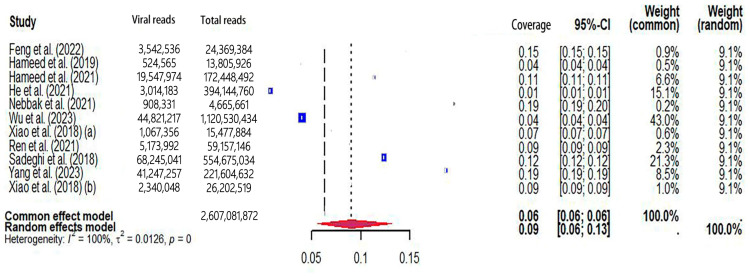
The viral coverage of mosquito viromes in pools of mosquitoes of mixed genera. A total of 11 reported the viral coverage of mosquito viromes in mosquito pools of mixed genera [17,19,41,42,44,45,46,48,50,51,52]. (a) [17] and (b) [52] were to distinguish different studies conducted in the same year by the first author with the same name (Pengpeng Xiao).

**Figure 3:**
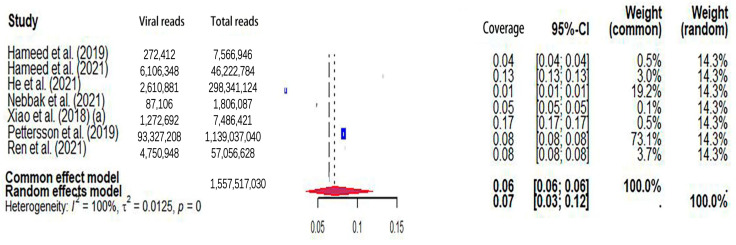
The viral coverage of mosquito viromes in pools of *Culex* (genus of mosquitoes). A total of 7 reported the viral coverage of mosquito viromes in mosquito pools of *culex* [17,42,44,45,46,49,50]. Note: “(a)” indicates two studies with the same first author and one of two studies performed in the same year [17].

**Figure 4:**
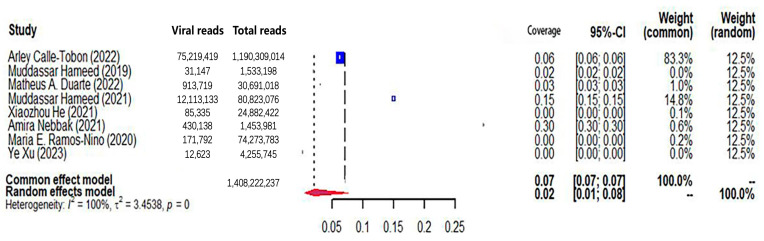
The viral coverage of mosquito viromes in pools of *Aedes* (genus of mosquitoes). A total of 8 reported the viral coverage of mosquito viromes in mosquito pools of *Aedes* [40,42,43,44,45,46,47,53].

**Figure 5:**
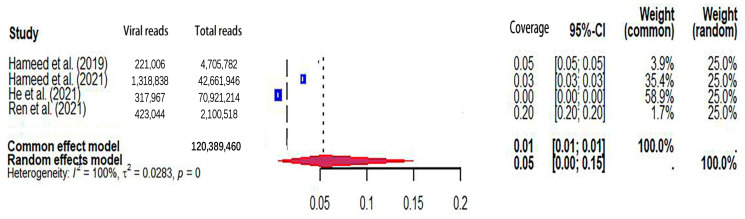
The viral coverage of mosquito viromes in pools of *Anopheles* (genus of mosquitoes). A total of 4 reported the viral coverage of mosquito viromes in mosquito pools of *Anopheles* [42,44,45,50].

The pooled viral coverage of mosquito viromes in mosquito pools of mixed genera was 8.99% (95% CI: 5.57–13.14%) (Figure 2). Sensitivity analyses employing the GLMM with logit transformation (Figure S1) revealed a slightly lower pooled coverage of 7.76% (95%CI: 5.29–11.25%) in mosquito pools of mixed genera. In *Culex* pools, the pooled viral coverage stood at 7.09% (95%CI: 3.44–11.91%), demonstrating statistical significance and high heterogeneity across studies (Figure 3). Following the application of the GLMM with logit transformation, the pooled viral coverage in *Culex* pools decreased to 5.98% (95%CI: 3.39–10.36%) (Figure S2). *Aedes* pools exhibited a pooled coverage of 2.11% (95%CI: 0.58–7.66%), with notable heterogeneity (Figure 4). Sensitivity analyses indicated a pooled viral coverage of 2.24% (95%CI: 1.38–3.60%) in mosquito pools of *Aedes* (Figure S3). In *Anopheles* pools, the pooled coverage was 5.28% (95%CI: 0.45–14.93%), accompanied by remarkable heterogeneity (Figure 5). Sensitivity analyses utilizing the GLMM with logit transformation (Figure S4) demonstrated a decrease in the pooled viral coverage of mosquito viromes in *Anopheles* pools to 3.53% (95%CI: 0.66–16.71%) (Figure S4).

### 3.4. Subgroup Analysis

An in-depth exploration of the viral coverage of mosquito viromes in mosquito pools spanning mixed genera, *Culex*, *Aedes*, and *Anopheles* unveiled a significant enhancement in virome coverage with the incorporation of saline buffer, medium, or antibiotics during the pre-treatment of mosquito samples (Table 3). With post-treatment with saline buffer, medium, and antibiotics, the viral coverage of mosquito viromes surged to 11.82% (95%CI: 10.88–12.78%) in the mixed genus, 10.40% (95%CI: 6.51–16.61%) in the *Culex* genus, 14.99% (95%CI: 14.98–15.00%) in the *Aedes* genus, and 8.01% (95%CI: 1.21–19.98%) in the *Anopheles* genus. The removal of the host genome before RNA library construction emerged as a pivotal factor influencing the heterogeneity of mosquito viromes coverage across all genera (Table 3). Notably, the removal of the host genome post-RNA extraction before RNA library construction led to a substantial increase in the pooled viral coverage of mosquito viromes in mixed genera pools (11.13%, 95% CI: 7.91–14.83%), *Culex* pools (8.66%, 95% CI: 5.11–13.14%), *Aedes* pools (8.69%, 95% CI: 2.80–26.98%), and *Anopheles* pools (8.01%, 95% CI: 1.21–19.98%).

Diverse bioinformatics pipelines (databases) exhibited no discernible impact on the viral coverage of mosquito viromes in mosquito pools of mixed genera and *Aedes* (*p* > 0.05). Similarly, varying sequencing read-lengths did not influence the viral coverage of mosquito viromes in the *Aedes* genus (*p* > 0.05). However, geographical region (continents) did influence the viral coverage of mosquito viromes in the *Aedes* genus (*p* < 0.001). Subgroup analysis of viral virome coverage in mixed genera pools highlighted climate zones as a significant heterogeneous factor (*p* < 0.05). In tropical climates, the viral coverage of mosquito viromes in the *culex*, *Aedes,* and *Anopheles* pools was 14.99% (95%CI: 11.70–19.19%), 1.32% (95%CI: 0.19–9.26%), and 8.01% (95%CI: 1.21–19.98%), respectively. In subtropical climates, the viral coverage of mosquito viromes in the *Culex* and *Aedes* pools was 5.25% (95%CI: 3.24–8.50%) and 7.24% (95%CI: 1.58–33.15%). In temperate climates, the viral coverage of mosquito viromes in *culex*, *Aedes,* and *Anopheles* pools was 2.68% (95%CI: 0.30–23.97%), 0.34% (95%CI: 0.34–0.35%), and 0.45% (95%CI: 0.45–0.45%), respectively. Detailed results of subgroup analysis are provided in Table 3.

### 3.5. Viral Spectrum Carried by Mosquitoes (Family Level)

Metagenomic analysis reveals a predominant presence of viruses carried by mosquitoes belonging to *Flaviviridae*, *Phenuiviridae*, *Circoviridae*, *Iflaviridae*, *Luteoviridae*, *Orthomyxoviridae*, *Rhabdoviridae*, *Solemoviridae*. Within the *Culex* genus, the primary virus families include *Flaviviridae*, *Circoviridae*, *Iflaviridae*, *Luteoviridae*, *Reoviridae*, and *Bunyaviridae*. Similarly, the *Aedes* genus is characterized by the prevalence of *Flaviviridae*, *Phenuiviridae*, *Iflaviridae*, and *Rhabdoviridae*, and the *Anopheles* genus predominantly harbors *Flaviviridae*, *Circoviridae*, *Orthomyxoviridae*, and *Genomoviridae* at the family level.

### 3.6. Publication Bias

Linear regression tests for funnel plot asymmetry showed that the likelihood of publication bias was relatively low in mosquito pools of mixed genera (t = 0.89, df = 9, *p* = 0.40), *Culex* (t = −0.11, df = 5, *p* = 0.92), *Aedes* (t = −0.25, df = 6, *p* = 0.81), and *Anopheles* (t = 1.77, df = 2, *p* = 0.22). Detailed funnel plots from meta-analyses, as depicted in Figures S5–S8, offer insights into the coverage of mosquito viromes within the mixed genera, *Culex*, *Aedes,* and *Anopheles*.

## 4. Discussion

Mosquitoes present a significant threat to public health due to their role in transmitting viral diseases. Metagenomic approaches have provided fresh insights into the intricate and diverse array of viruses carried by mosquitoes. These insights are crucial for active pathogen surveillance and for effectively addressing emerging and re-emerging infectious diseases [14,19,54,55]. The advancement of mNGS technologies has notably enhanced research in viral ecology, particularly focusing on RNA viruses [56,57]. These novel RNA viruses have been unearthed through viromic studies conducted on hosts, environments, and vectors. Mosquito viromic studies offer a novel perspective on understanding the potential involvement of vectors in viral ecology. Therefore, it is imperative to further refine the methodology of meta-viromic sequencing technology and broaden its application to enhance mosquito-borne virus surveillance. This knowledge expansion has not only deepened our comprehension of the viral realm but has also played a pivotal role in the initiation of the ‘Global Virome Project’ [58,59], which motivated the inception of our study.

The outcomes of our study revealed significant variations in the coverage of mosquito viromes across the three mosquito groups—*Culex*, *Aedes*, and *Anopheles*. The pooled coverage of *Culex* pools stood at 7.09% (95% CI: 3.44–11.91%), whereas *Aedes* pools exhibited a lower coverage of 2.11% (95% CI: 0.58–7.66%). It is important to note that the mosquito genera *Culex*, *Aedes*, and *Anopheles* exhibit distinct major virus spectra at the family level. These distinctions may be linked to the biological characteristics of the vectors, their feeding habits, and geographical distribution, among other factors [16,20,54]. Our study also revealed differences in geographic regions (continents) as a factor of heterogeneity affecting the viral coverage of mosquito viromes in the *Aedes* genus. This difference would be more biologically meaningful when reflected at the species level. For instance, results from a previous study analyzing the metagenomic viromes of *Culex tritaeniorhynchus* mosquitoes from Kenya and China revealed differences in the composition of mosquito viromes [54]. This study was conducted only at the genus level of mosquitoes, and in the future, we expect to be able to explore the mosquito viromes at the species level of mosquitoes, which will make the results of the study more precise and relevant.

With pre-treatment of mosquito samples with saline buffer/medium plus antibiotics, the viral coverage of mosquito viromes increased to 10.40% (95% CI: 6.51–6.51%) for *Culex*, 14.99% (95% CI: 14.98–15.00%) for *Aedes*, and 8.01% (95% CI: 1.21–19.98%) for *Anopheles.* That is, this step is necessary when performing meta-viromic sequencing of mosquitoes. Upon the removal of the host genome post-RNA extraction before RNA library construction, the pooled coverage of mosquito viromes in mixed genera mosquito pools surged to 11.13% (95% CI: 7.91–14.83%). The coverage percentages for *Culex*, *Aedes*, and *Anopheles* also saw significant increases, emphasizing the critical nature of removing the host genome, particularly rRNA. The elimination of rRNA, as detailed in the original literature referenced in this study, was primarily achieved using Ribo-Zero’s Illumina Stranded Total RNA Prep kit. This process effectively eliminates rRNA from total RNA, fragments the remaining RNA chemically, and randomizes primers for reverse transcription.

The bioinformatics pipelines utilized in the subgroup analysis of *Culex* and *Anopheles* mosquitoes were found to be similar, leading to no distinct differentiation. However, the study’s outcomes highlighted that the bioinformatics pipelines did not contribute to the heterogeneity in the coverage of mosquito viromes observed through meta-viromic sequencing, encompassing mixed genera and *Aedes* mosquitoes. Additionally, we delved into the subgroup variable “sequencing read-length” by examining the virome coverage in *Aedes* mosquitoes, revealing no statistically significant differences. This lack of disparity could be attributed to the varying construction methodologies of genomic libraries across different next-generation platforms [40,41,51,52]. Nevertheless, the overarching objective remains consistent: to procure high-quality sequences of the appropriate size for sequencing and to derive precise comparative results through standardized procedures [60].

It is important to note that the criteria for virus identification across the diverse original studies included in this meta-analysis were not uniform. Varying thresholds for genome coverage, the number of virus mapping contigs or reads, as well as nucleotide or amino acid sequence identity scores were employed in different studies for virus identifications. Despite these discrepancies, our bioinformatics analysis pipeline accounted for these heterogeneities and did not identify them as significant factors post-analysis. However, this pertains solely to the coverage of mosquito viromes and does not facilitate the quantification of the identified virus species across different studies. Hence, standardizing criteria to the greatest extent possible is imperative for meaningful cross-study comparisons.

So far, no meta-analysis focusing on the coverage of mosquito viromes derived from meta-viromic sequencing has been conducted. The urgent necessity to comprehend the pathogen-identifying potential of meta-viromic sequencing in mosquito-borne virus surveillance underscores the novelty of our study. Our assessment of mosquito virome coverage has shed light on the current landscape of meta-viromic sequencing technology in mosquito-borne virus surveillance, emphasizing its pivotal role in pathogen detection. Notably, our systematic review and meta-analysis unveiled that factors such as host genome removal prior to RNA library construction, distinct climatic zones, and the mosquito genera composition contribute to the heterogeneity in mosquito virome coverage. These represent the key innovative discoveries gleaned from our comprehensive analysis.

Some limitations of our study need addressing. Firstly, it is crucial to note the considerable unexplained heterogeneity in the subgroup analysis, which complicates the interpretation and utility of the combined-effect estimate. Alongside the identified heterogeneity factors, the coverage of the mosquito viromes may have been influenced by various factors such as the blood-sucking habits of female mosquitoes, the micro-ecology fostering microbial diversity, the breadth of the mosquito’s diet encompassing plants and blood, contaminants in their resting environmental substrates, and other variables [61,62,63,64,65]. Furthermore, significant differences exist in the virome composition among mosquito species due to their distinct biological habitats, exposing them to varying environmental viromes [17,66,67]. Secondly, the inclusion of a limited number of studies, particularly in the pooled analysis of the genus *Anopheles*, indicated insufficient evidence. The relatively scarce scientific publications on the *Anopheles* virome underscore the gaps in our understanding. In the present study, the number of mosquitoes per pool in almost all mosquito pools was above 100. There is not a sufficient number of studies to support us in exploring whether mosquito pool size is a factor in the heterogeneity of mosquito virome coverage. It is vital to mention that the present study is very broad in exploring whether mosquito virome coverage is dependent on geographic region based only on continent-level divisions. Based on the amount of primary literature included in this study and the limitations of the level of detail of the geographic information, we were unable to analyze the above questions at a more geographic level. In addition, it is important to mention that for the 16 original publications included in this study, most of the meta-viromic sequencing of mosquito viromes has been at the mixed-genus or single-genus level. From a biological point of view, concerning arboviruses, the identification of pathogens on the genus level is overly broad. We expect that more scholars will explore this in the future, to the extent that we can explore the above issues at the mosquito species level. It is anticipated that future discussions will incorporate additional studies on the application of meta-viromic sequencing technology in mosquito-borne virus surveillance.

## 5. Conclusions

Our findings indicate differences in virome coverage among various mosquito genera, and the meta-viromic sequencing process also impacts mosquito-vector virome coverage, particularly through pre-treatment of mosquito samples with saline buffer/medium/antibiotics before DNase/RNase treatment and RNA extraction and the removal of host genomes before RNA library construction, all of which significantly enhance mosquito virome coverage. Overall, our study offers a comprehensive analysis of mosquito-vector viromes obtained through meta-viromic sequencing, conducted via systematic review and meta-analysis. The aim is to provide a theoretical basis for optimizing meta-viromic sequencing methodology, expanding its applications, and improving the implementation of mosquito-borne virus surveillance.

## Figures and Tables

**Table 1:** Summary of basic characteristics of the viral coverage of mosquito viromes in mosquito pools of mixed *Culex*, *Aedes*, *Anopheles*, and mixed genera.

Study	Levels of Mosquito Genus	Viral Reads	Total Reads	Pre-Treatment of Mosquito Samples (Before DNase/RNase Treatment and RNA Extraction)	Removal of the Host Genome (Pre-Library Sequencing)	Sequencing Read-Length	Bioinformatics Pipeline (Database)
Arley Calle-Tobo’n (2022) [40]	*Aedes*	75,219,419	1,190,309,014	No ^a^	Yes	<150 bp	Custom database
Guanrong Feng (2022) [41]	Mixed genera	3,542,536	24,369,384	Saline buffer	Yes	150~300 bp	NCBI database
Muddassar Hameed (2019) [42]	*Culex*	272,412	7,566,946	Saline buffer	Yes	150~300 bp	NCBI database
	*Aedes*	31,147	1,533,198				
	*Anopheles*	221,006	4,705,782				
	Mixed genera	524,565	13,805,926				
Matheus A. Duarte (2022) [43]	*Aedes*	913,719	30,691,018	Saline buffer	No	150~300 bp	NCBI database
Muddassar Hameed (2021) [44]	*Culex*	6,106,348	46,222,784	Medium + Antibiotics	Yes	150~300 bp	NCBI database
	*Aedes*	12,113,133	80,823,076				
	*Anopheles*	1,318,838	42,661,946				
	Mixed genera	19,547,974	172,448,492				
Xiaozhou He (2021) [45]	*Culex*	2,610,881	298,341,124	No ^a^	No	150~300 bp	NCBI database
	*Anopheles*	317,967	70,921,214				
	*Aedes*	85,335	24,882,422				
	Mixed genera	3,014,183	394,144,760				
Amira Nebbak (2021) [46]	*Aedes*	430,138	1,453,981	Medium	Yes	150~300 bp	NCBI database
	*Culex*	87,106	1,806,087				
	Mixed genera	908,331	4,665,661				
Maria E. Ramos-Nino (2020) [47]	*Aedes*	171,792	74,273,783	No ^a^	No	<150 bp	NCBI database
Qin Wu (2023) [48]	Mixed genera	44,821,217	1,120,530,434	No ^a^	No	150~300 bp	Custom database
Pengpeng Xiao (2018) (a) [17]	*Culex*	1,272,692	7,486,421	Saline buffer	Yes	150~300 bp	NCBI database
	Mixed genera	1,067,356	15,477,884				
John H.-O. Pettersson (2019) [49]	*Culex*	93,327,208	1,139,037,040	Saline buffer + Antibiotics	Yes	150~300 bp	NCBI database
Nanjie Ren (2021) [50]	*Culex*	4,750,948	57,056,628	Medium	Yes	NA *	NCBI database
	*Anopheles*	423,044	2,100,518				
	Mixed genera	5,173,992	59,157,146				
Mohammadreza Sadeghi (2018) [19]	Mixed genera	68,245,041	554,675,034	Medium + Antibiotics	Yes	150~300 bp	NCBI database
Xiaojing Yang (2023) [51]	Mixed genera	41,247,257	221,604,632	Saline buffer	Yes	150~300 bp	Custom database
Pengpeng Xiao (2018) (b) [52]	Mixed genera	2,340,048	26,202,519	Saline buffer	Yes	150~300 bp	NCBI database
Ye Xu (2023) [53]	*Aedes*	12,623	4,255,745	Saline buffer	No	<150 bp	Custom database

^a^ No medium/saline buffer/antibiotics were added to the pre-treatment of mosquito samples in the original literature. * Sequencing read-lengths were not provided in the original literature or were not available. (a) [17] and (b) [52] were to distinguish different studies conducted in the same year by the first author with the same name (Pengpeng Xiao).

**Table 2:** Quality Review Scores.

Study	Research Question	Sampling Location	Temperature Description	Sampling Method	Pre-Treatment of Mosquito Samples (Before DNase/RNase Treatment and RNA Extraction)	Nucleic Acid Extraction (RNA)	Removal of the Host Genome (Pre-Library Sequencing)	Sequencing Platforms
Arley Calle-Tobo’n (2022) [40]	3	3	3	3	1	3	3	3
Guanrong Feng (2022) [41]	3	2	2	2	3	3	3	2
Muddassar Hameed (2019) [42]	3	2	2	2	3	3	3	2
Matheus A. Duarte (2022) [43]	3	2	2	2	3	3	1	3
Muddassar Hameed (2021) [44]	3	2	2	3	3	3	3	2
Xiaozhou He (2021) [45]	3	2	2	3	1	3	1	3
Amira Nebbak (2021) [46]	3	3	1	3	3	3	3	2
Maria E. Ramos-Nino (2020) [47]	2	3	1	2	1	3	1	3
Qin Wu (2023) [48]	3	2	2	3	1	3	1	3
Pengpeng Xiao (2018) (a) [17]	3	2	2	1	3	3	3	2
John H.-O. Pettersson (2019) [49]	2	3	1	2	3	3	3	3
Nanjie Ren (2021) [50]	3	3	1	3	3	3	3	3
Mohammadreza Sadeghi (2018) [19]	3	2	1	2	3	3	3	3
Xiaojing Yang (2023) [51]	3	2	2	2	3	3	3	3
Pengpeng Xiao (2018) (b) [52]	3	2	2	1	3	3	3	2
Ye Xu (2023) [53]	3	3	2	1	3	3	1	3

Notes: (a) and (b) were to distinguish different studies conducted in the same year by the first author with the same name (Pengpeng Xiao).

**Table 3:** Subgroup analysis of the viral coverage of mosquito viromes in mosquito pools of mixed genera, *Culex*, *Aedes*, and *Anopheles*.

Variables for Subgroup Analysis		Number of Studies	Pooled Estimate (%, 95% CI)	*p*-Value
Pre-treatment of mosquito samples (before DNase/RNase treatment and RNA extraction)				
Mixed genera	Saline buffer/Medium	7	10.94 (6.87–15.82)	0.002
	Saline buffer/Medium +Antibiotics *	2	11.82 (10.88–12.78)	
	No ^a^	2	2.07 (0.11–6.41)	
*Culex*	Saline buffer/Medium	4	7.04 (3.61–13.75)	<0.001
	Saline buffer/Medium +Antibiotics *	2	10.40 (6.51–16.61)	
	No ^a^	1	0.88 (0.87–0.88)	
*Aedes*	Saline buffer/Medium	4	2.70 (0.42–17.18)	0.004
	Saline buffer/Medium +Antibiotics *	1	14.99 (14.98–15.00)	
	No ^a^	3	0.79 (0.10–6.14)	
*Anopheles*	Saline buffer/Medium/Antibiotics *	3	8.01 (1.21–19.98)	0.015
	No ^a^	1	0.45 (0.45–0.45)	
Removal of the host genome (before RNA library construction)				
Mixed genera	Yes	9	11.13 (7.91–14.83)	0.002
	No	2	2.07 (0.11–6.41)	
*Culex*	Yes	6	8.66 (5.11–13.14)	<0.001
	No	1	0.88 (0.87–0.88)	
*Aedes*	Yes	4	8.69 (2.80–26.98)	0.002
	No	4	0.51 (0.16–1.64)	
*Anopheles*	Yes	3	8.01 (1.21–19.98)	0.015
	No	1	0.45 (0.45–0.45)	
Bioinformatics pipeline (database)				
Mixed genera	NCBI database	9	8.75 (5.15–13.19)	0.855
	Custom database	2	10.12 (0.70–28.53)	
*Aedes*	NCBI database	6	2.44 (0.51–11.62)	0.737
	Custom database	2	1.37 (0.07–27.44)	
Sequencing read-length				
*Aedes*	<150 bp	3	0.76 (0.09–6.09)	0.214
	150~300 bp	5	3.91 (0.84–18.24)	
Climatic zones				
Mixed genera	Subtropical	6	12.29 (7.62–17.89)	<0.001
	Tropical	4	7.56 (4.73–10.98)	
	Temperate	1	0.76 (0.76–0.77)	
*Culex*	Subtropical	3	5.25 (3.24–8.50)	<0.001
	Tropical	2	14.99 (11.70–19.19)	
	Temperate	2	2.68 (0.30–23.97)	
*Aedes*	Subtropical	3	7.24 (1.58–33.15)	<0.001
	Tropical	4	1.32 (0.19–9.26)	
	Temperate	1	0.34 (0.34–0.35)	
*Anopheles*	Tropical	3	8.01 (1.21–19.98)	0.015
	Temperate	1	0.45 (0.45–0.45)	

^a^ No medium/saline buffer/antibiotics were added to the pre-treatment of mosquito samples in the original literature. * Saline buffer or medium or saline buffer + antibiotics or medium + antibiotics.

## Supplementary Materials

The following supporting information can be downloaded at: https://www.mdpi.com/article/10.3390/microorganisms12091899/s1, Figure S1: Sensitivity analyses of the viral coverage of mosquito viromes in pools of mosquitoes of mixed genera; Figure S2: Sensitivity analyses of the viral coverage of mosquito viromes in pools of *Culex* (genus of mosquitoes); Figure S3: Sensitivity analyses of the viral coverage of mosquito viromes in pools of *Aedes* (genus of mosquitoes); Figure S4: Sensitivity analyses of the viral coverage of mosquito viromes in pools of *Anopheles* (genus of mosquitoes); Figure S5: Funnel plot of the publication bias evaluation of the viral coverage of mosquito viromes in pools of mosquitoes of mixed genera; Figure S6: Funnel plot of the publication bias evaluation of the viral coverage of mosquito viromes in pools of *Culex* (genus of mosquitoes); Figure S7: Funnel plot of the publication bias evaluation of the viral coverage of mosquito viromes in pools of *Aedes* (genus of mosquitoes); Figure S8: Funnel plot of the publication bias evaluation of the viral coverage of mosquito viromes in pools of *Anopheles* (genus of mosquitoes); Table S1: Details of 16 included original studies. Supplementary File S1: Embase, Web of science, Medline (via PubMed), Science-Direct, and Scopus research.
