# Supplementary material for: An Exploration of the Viral Coverage of Mosquito Viromes Using Meta-Viromic Sequencing: A Systematic Review and Meta-Analysis
Source: Microorganisms. 2024 Sep 14;12(9):1899. doi: 10.3390/microorganisms12091899 (PMC11434593; doi:10.3390/microorganisms12091899)
Supplement: Supplementary file 1 [file microorganisms-12-01899-s001.zip › Supplementary File S1.pdf]

Supplementary File S1: Embase, Web of science, Medline (via PubMed), Science-Direct, and Scopus research

**Embase**	
1	exp meta-viromic sequencing.mp. or metagenomic sequencing.mp. or metatranscriptomics. mp
2	exp mosquito*.mp.
3	1 and 2
**Web of science**	
1	meta-viromic sequencing [title, abstract and indexing]
2	metagenomic shotgun sequencing OR metagenomic sequencing [title, abstract and indexing]
3	metagenomic sequencing OR meta-viromic sequencing [Keywords Plus]
4	1 OR 2 OR 3
5	Mosquito [Abstract]
6	Virome [title, abstract and indexing]
7	4 and 5 and 6
**Medline (via PubMed)**	
1	(metagenomic next-generation sequencing OR metagenomic shotgun sequencing OR) [MeSH Terms]
2	(meta-viromic sequencing) [All fields]
3	1 OR 2
4	Mosquito * [Title/Abstract]
5	3 and 4
**Science-Direct**	
1	meta-viromic sequencing OR metagenomic sequencing OR metagenomic shotgun sequencing) [Title, abstract, keywords]
2	Mosquito [Title, abstract, keywords]
3	Virome [Title, abstract, keywords]
4	1 and 2 and 3
**Scopus**	
1	(TITLE-ABS-KEY (meta-viromic AND sequencing) OR TITLE-ABS-KEY (metagenomic AND sequencing) OR TITLE-ABS-KEY (metagenomic AND next-generation AND sequencing) OR TITLE-ABS-KEY (metagenomic AND shotgun AND sequencing) AND TITLE-ABS-KEY (mosquito) AND ALL (virome))
