# Supplementary figures and images for: An Exploration of the Viral Coverage of Mosquito Viromes Using Meta-Viromic Sequencing: A Systematic Review and Meta-Analysis
Source: Microorganisms. 2024 Sep 14;12(9):1899. doi: 10.3390/microorganisms12091899 (PMC11434593; doi:10.3390/microorganisms12091899)

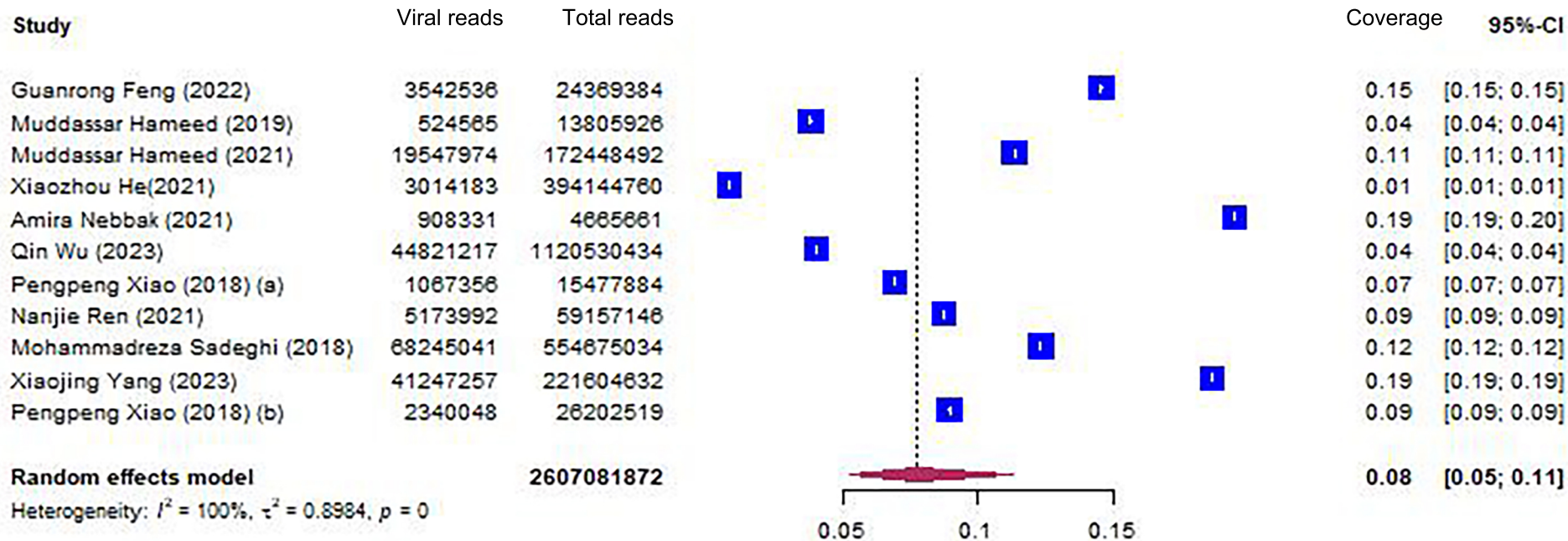

Supplement: Supplementary file 1 [file microorganisms-12-01899-s001.zip › Figure S1.pdf]

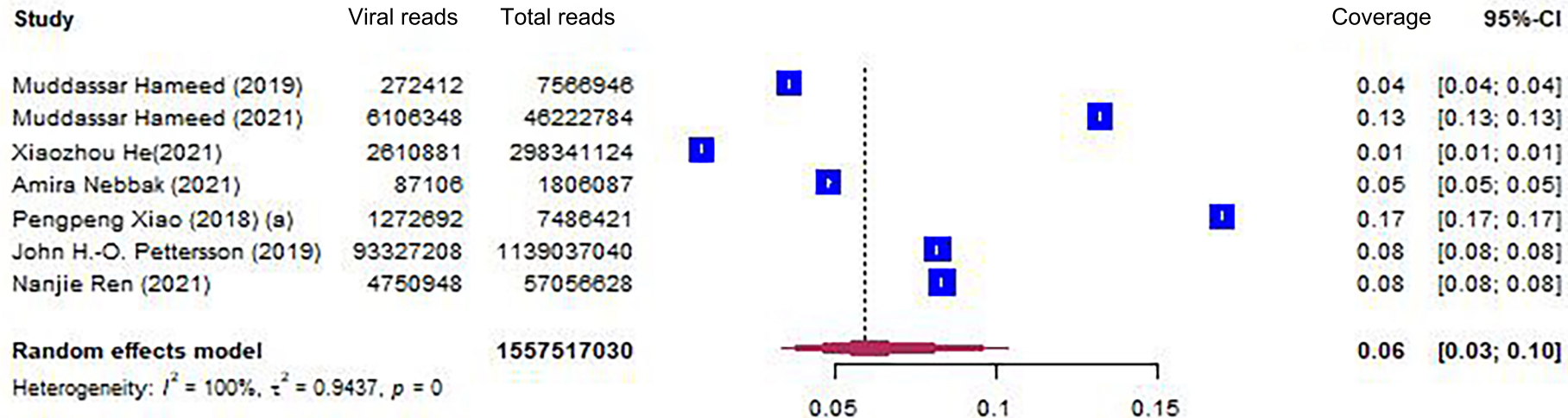

Supplement: Supplementary file 1 [file microorganisms-12-01899-s001.zip › Figure S2.pdf]

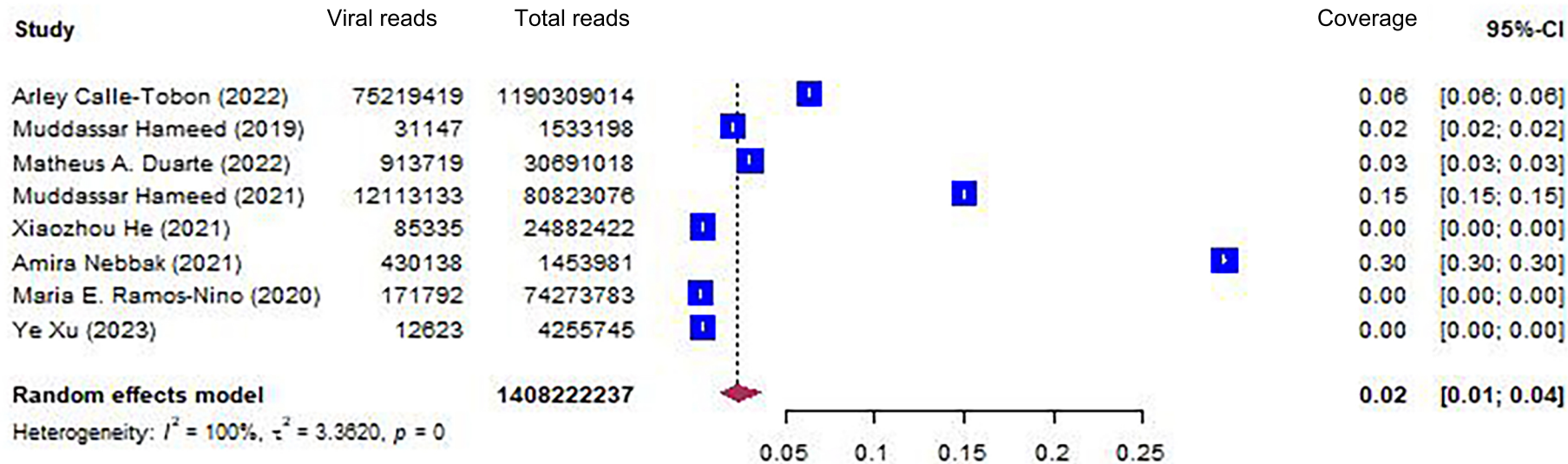

Supplement: Supplementary file 1 [file microorganisms-12-01899-s001.zip › Figure S3.pdf]

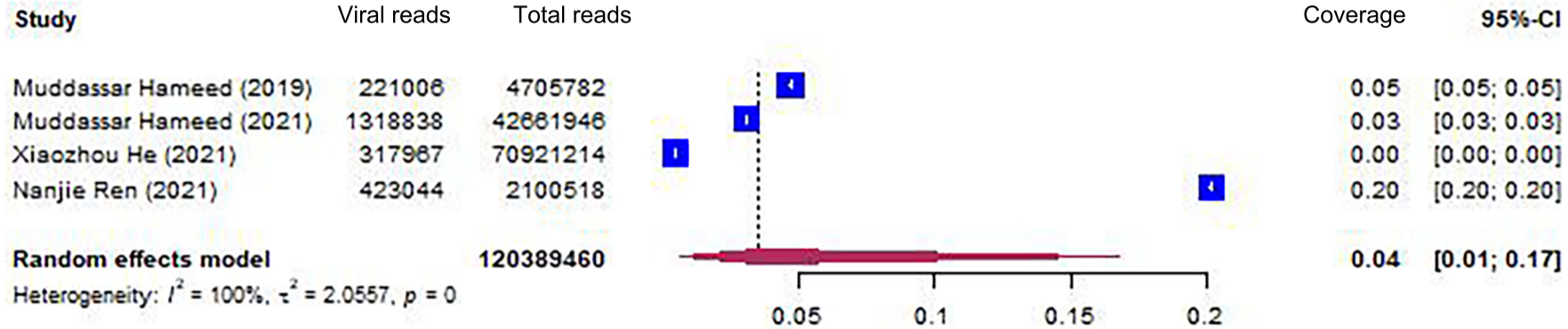

Supplement: Supplementary file 1 [file microorganisms-12-01899-s001.zip › Figure S4.pdf]

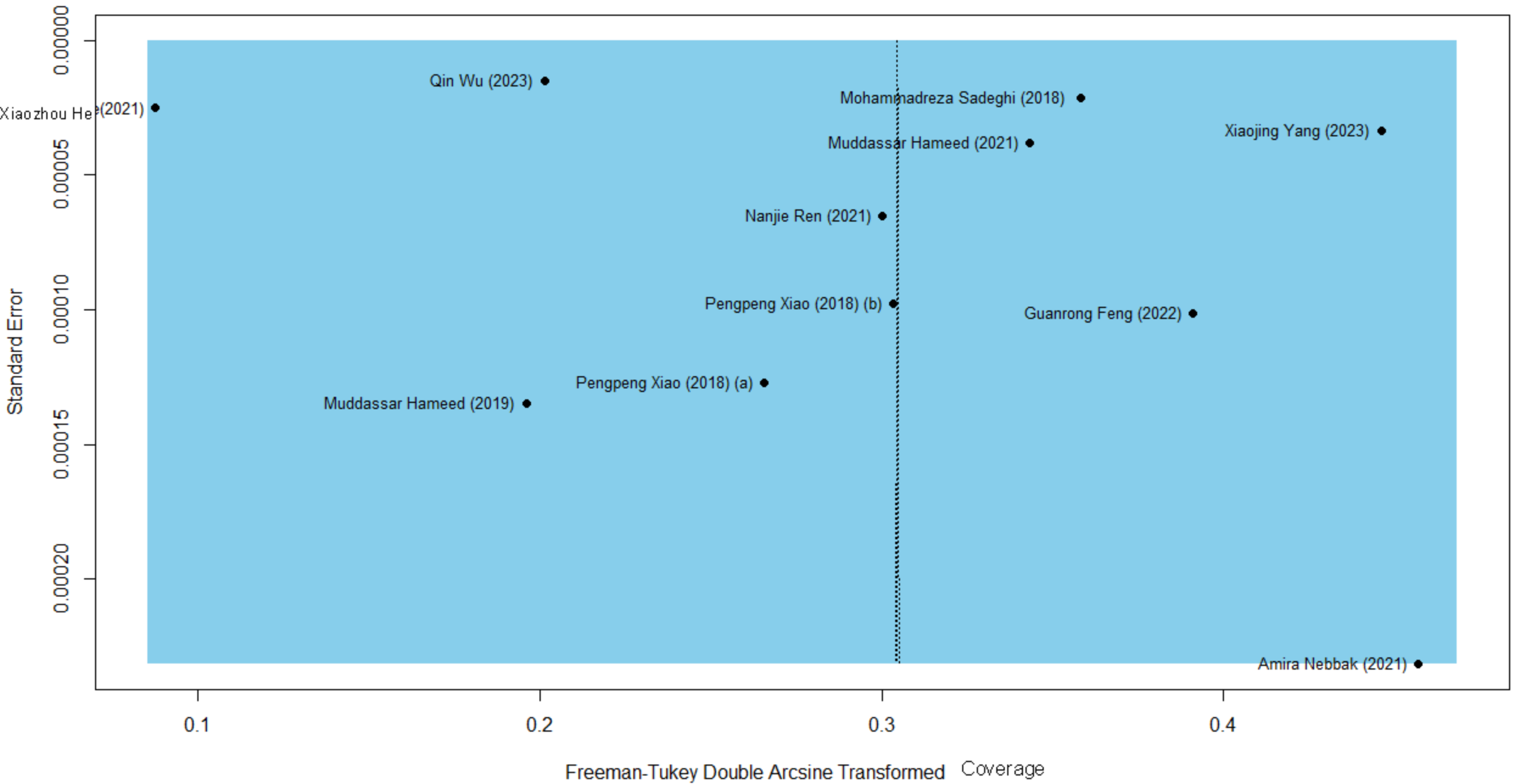

Supplement: Supplementary file 1 [file microorganisms-12-01899-s001.zip › Figure S5.pdf]

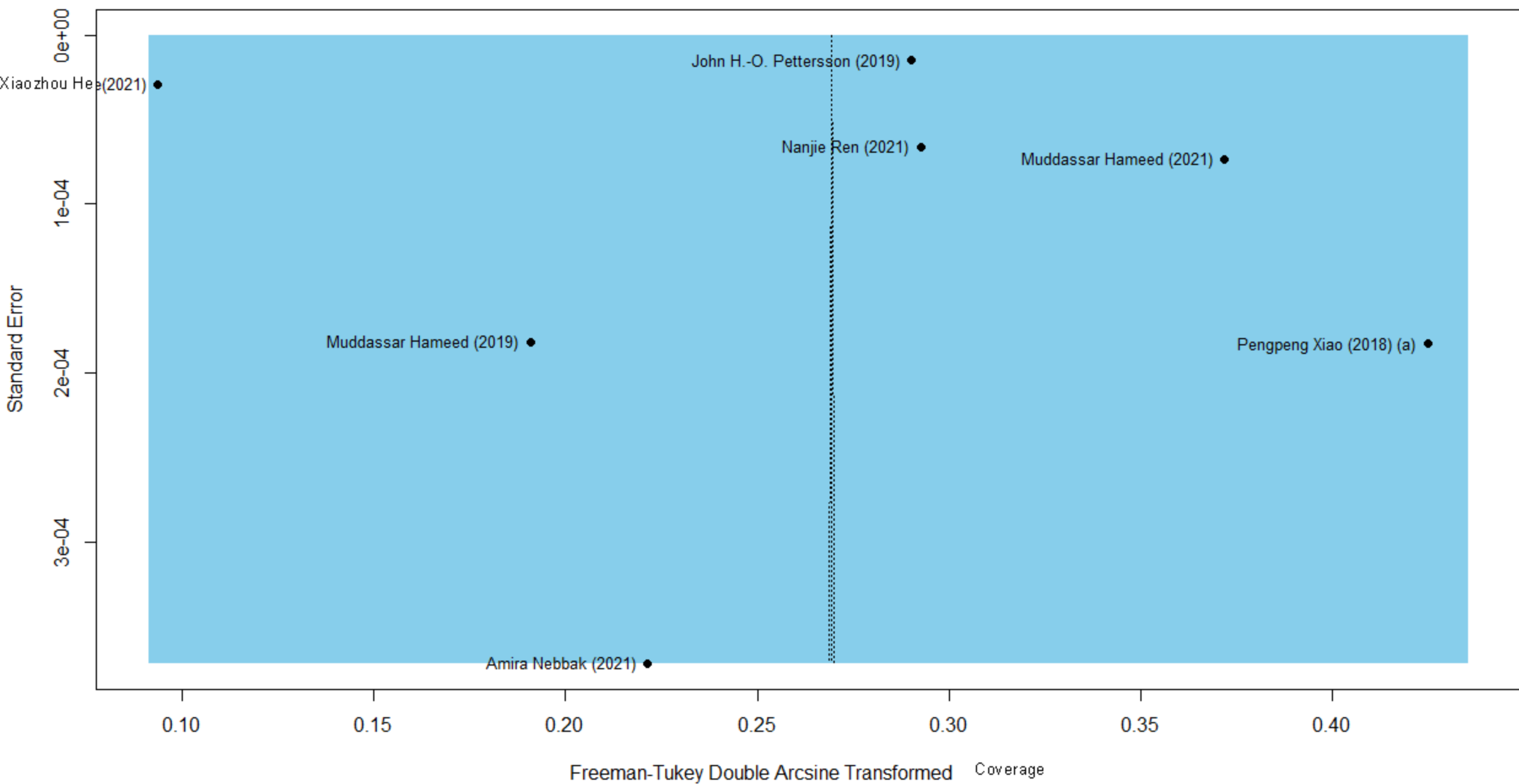

Supplement: Supplementary file 1 [file microorganisms-12-01899-s001.zip › Figure S6.pdf]

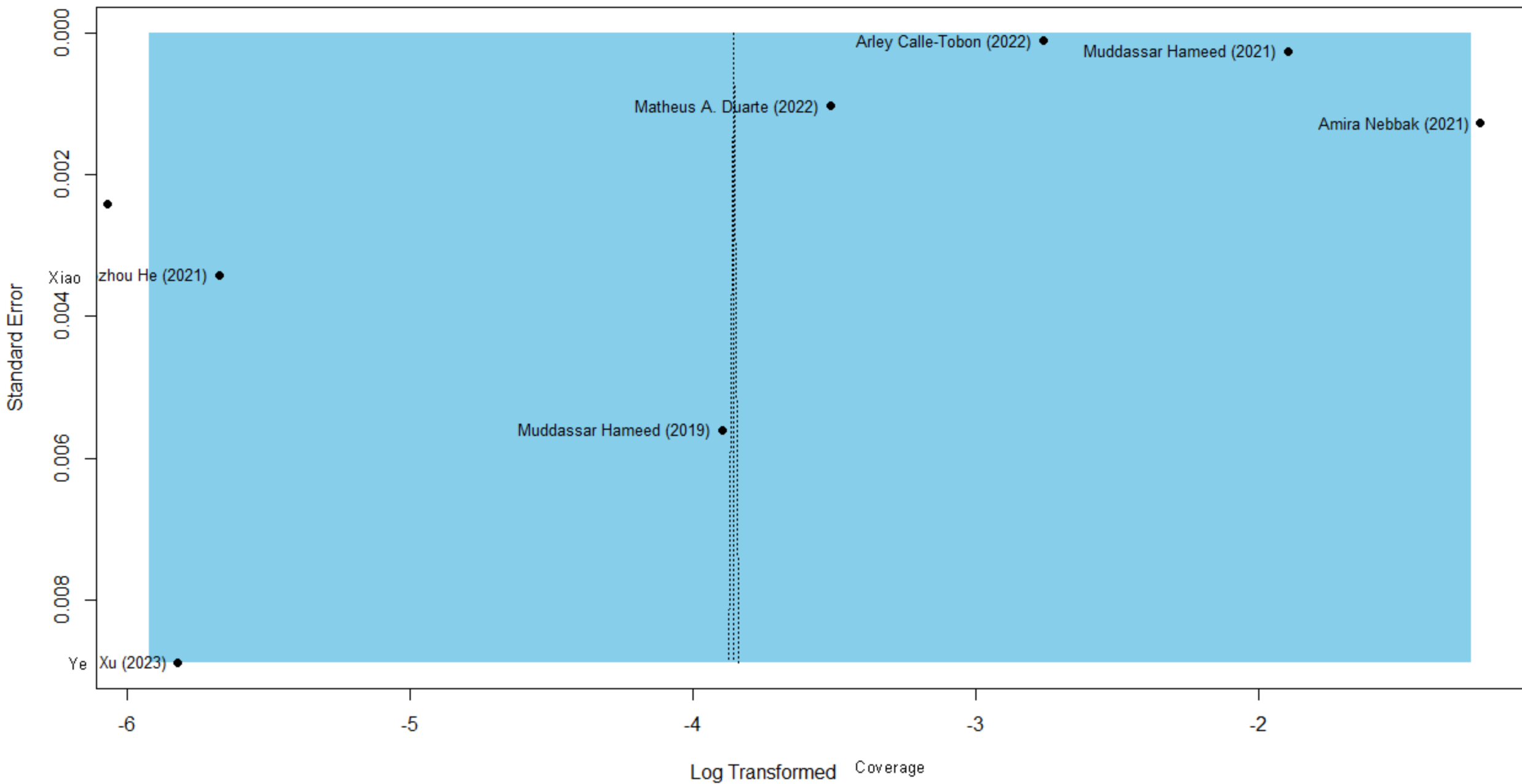

Supplement: Supplementary file 1 [file microorganisms-12-01899-s001.zip › Figure S7.pdf]

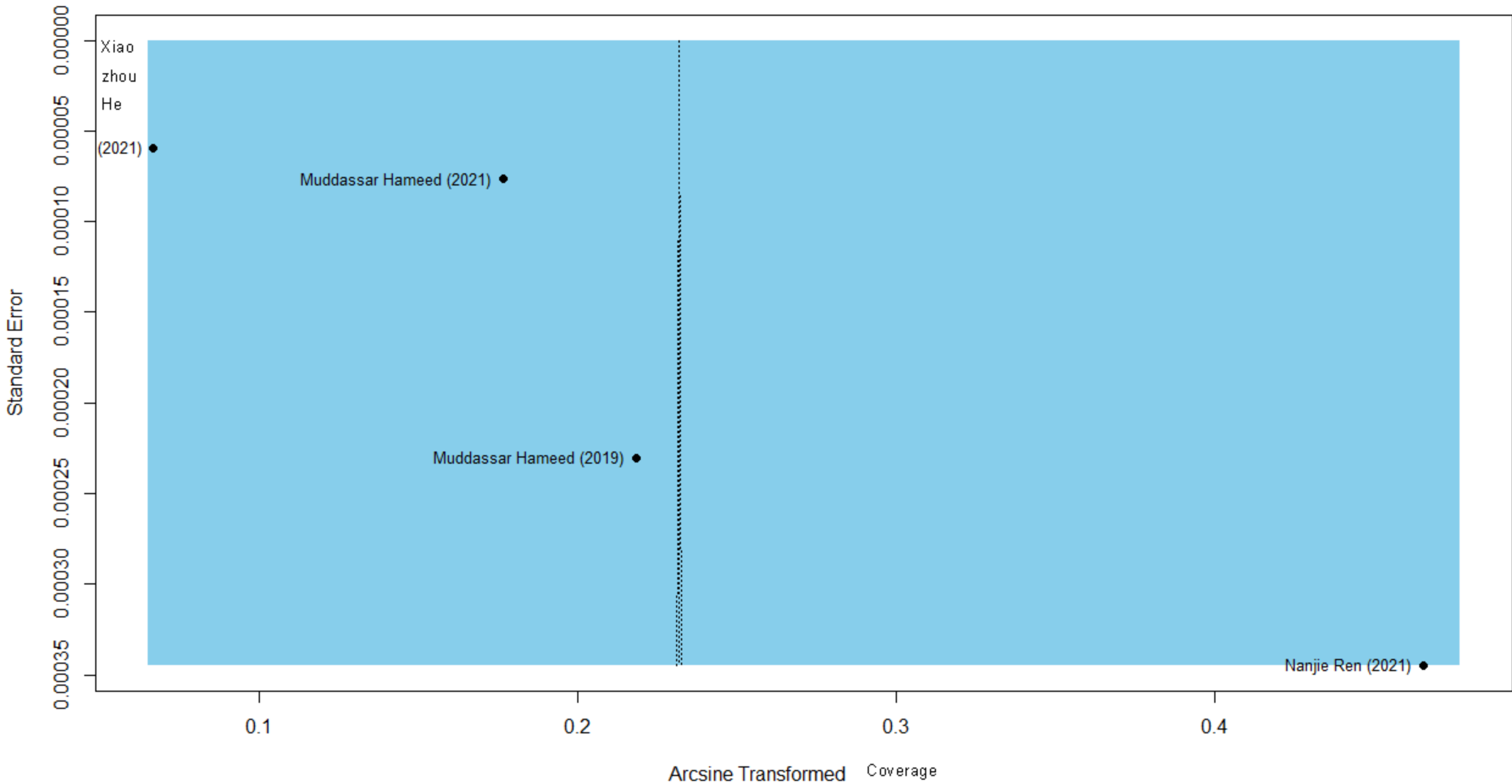

Supplement: Supplementary file 1 [file microorganisms-12-01899-s001.zip › Figure S8.pdf]
